# Revealing the therapeutic targets and molecular mechanisms of emodin-treated coronavirus disease 2019 via a systematic study of network pharmacology

**DOI:** 10.18632/aging.203098

**Published:** 2021-06-04

**Authors:** Hai-Xia Du, Jia-Qi Zhu, Jing Chen, Hui-Fen Zhou, Jie-Hong Yang, Hai-Tong Wan

**Affiliations:** 1 College of Basic Medical Science, Zhejiang Chinese Medical University, Hangzhou 310053, China; 2 College of Life Science, Zhejiang Chinese Medical University, Hangzhou 310053, China

**Keywords:** emodin, COVID-19, pathway, network pharmacology, molecular docking

## Abstract

Emodin has shown pharmacological effects in the treatment of infection with severe acute respiratory syndrome coronavirus-2, which leads to coronavirus disease 2019 (COVID-19). Thus, we speculated that emodin may possess anti-COVID-19 activity. In this study, using bioinformatics databases, we screened and harvested the candidate genes or targets of emodin and COVID-19 prior to the determination of pharmacological targets and molecular mechanisms of emodin against COVID-19. We discovered core targets for the treatment of COVID-19, including mitogen-activated protein kinase 1 (MAPK1), tumor protein (TP53), tumor necrosis factor (TNF), caspase-3 (CASP3), epidermal growth factor receptor (EGFR), vascular endothelial growth factor A (VEGFA), interleukin 1B (IL1B), mitogen-activated protein kinase 14 (MAPK14), prostaglandin-endoperoxide synthase 2 (PTGS2), B-cell lymphoma-2-like protein 1 (BCL2L1), interleukin-8 (CXCL8), myeloid cell leukemia-1 (MCL1), and colony stimulating factor 2 (CSF2). The GO analysis of emodin against COVID-19 mainly included cytokine-mediated signaling pathway, response to lipopolysaccharide, response to molecule of bacterial origin, developmental process involved in reproduction, and reproductive structure development. The KEGG results exhibited that the molecular pathways mainly included IL-17 signaling pathway, AGE-RAGE signaling pathway in diabetic complications, TNF signaling pathway, pertussis, proteoglycans in cancer, pathways in cancer, MAPK signaling pathway, NOD-like receptor signaling pathway, NF-kappa B signaling pathway, etc. Also, molecular docking results revealed the docking capability between emodin and COVID-19 and the potential pharmacological activity of emodin against COVID-19. Taken together, these findings uncovered the targets and pharmacological mechanisms of emodin for treating COVID-19 and suggested that the vital targets might be used as biomarkers against COVID-19.

## INTRODUCTION

The etiology of coronavirus disease 2019 (COVID-19) is caused by infection with severe acute respiratory syndrome coronavirus 2 (SARS-CoV-2), with patients experiencing fever, dry cough, and fatigue as common symptoms [1]. In comparison with other coronaviruses, SARS-CoV-2 shows lower pathogenesis but higher transmission capacity [2, 3]. To date, COVID-19 has been sweeping through many different geographical areas in a timescale of minutes and seconds [4, 5]. Indeed, the worldwide outbreak of the disease has emerged as a severe threat to public health, claiming many lives, and has come to be regarded as a global health emergency in January 2020 and a pandemic in March 2020, as declared by the World Health Organization (WHO) [6]. In light of these declarations and especially given the novelty of this condition, exploring and developing effective therapeutic drugs or vaccines is crucial, yet effective therapeutics are still limited in number even one year later.

Traditional Chinese medicine (TCM) has been trialed to treat COVID-19 and has achieved good curative effects [7]. Emodin (1, 3, 8-trihydroxy-6-methylanthraquinone) is an anthraquinone derivative from the roots and barks of *Rheum palmatum, Polygonum cuspidatum and Polygonum multiflorum* [8, 9], and has evident pharmacological benefits, supporting the inhibition of hepatitis B virus replication *in vitro* or *in vivo* [10], the anti-proliferation of lung cancer cells [11], and the regulation of immune response or inflammatory activities [12]. Previous works have reported on the inhibition of influenza A virus replication and pneumonia [13]. Herein, emodin is not only an inhibitor of the SNE-encoded 3a protein, but also blocks the interaction between S protein and angiotensin-converting enzyme 2 (ACE2) [14, 15]. Meanwhile, the latest research findings have indicated that SARS-CoV-2 infection is associated with an affinity of S proteins and ACE2 receptors [16]. However, to date, no exploration of the efficacy of emodin administration against COVID-19, especially its potential targets and mechanisms, has been performed.

Andrew L. Hopkins, a British pharmacologist, first introduced a new discipline based on systems biology [17], which came to be known as network pharmacology. This discipline focuses on the multi-targets regulation of signaling pathways. Importantly, it not only promotes the success of clinical trials of novel drugs, but also reduces expenses in drug development [18]. Molecular docking is an approach of drug-design that involves establishing the relationships between molecules and receptors [19], which contributes to the simulation of interactions between compounds and proteins; notably, this helps to enhance the credibility of network pharmacology.

**Figure 1:**
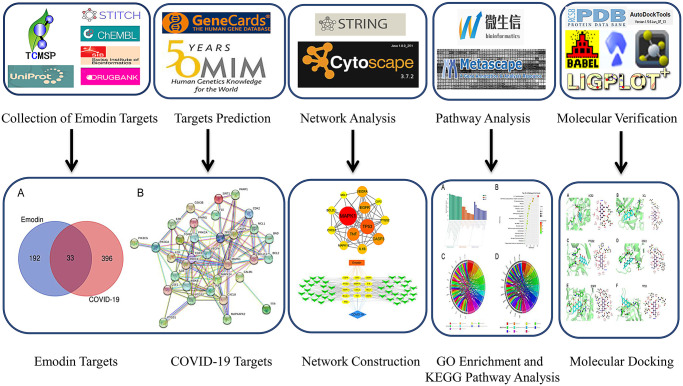
Flow diagram of network pharmacological research on emodin against COVID-19.

Collectively, to reveal biological targets and signaling pathways connected with the disease, an analysis of network pharmacology and molecular docking technology of emodin against COVID-19 was carried out. In this research (Figure 1), we first identified potential targets of emodin in COVID-19, and then investigated the intersection of the drug targets and genes associated with diseases. Additionally, a protein-protein interaction (PPI) network was constructed to reveal interactions among proteins. Gene Ontology (GO) and Kyoto Encyclopedia of Genes and Genomes (KEGG) enrichment analysis were performed based on core targets. Eventually, we performed molecular docking to verify the interactions between emodin and its selected targets. Our findings may help to elucidate the pharmacological mechanism of emodin against COVID-19 and increase the possibility of developing novel therapeutics for this condition.

## MATERIALS AND METHODS

### Screening of candidate genes associated with emodin

To obtain potential pharmacological targets associated with emodin, online databases were used. These databases include Traditional Chinese Medicine Systems Pharmacology (TCMSP, https://tcmspw.com/tcmsp.php, version 2.3), Drugbank (https://www.drugbank.ca/, updated September 26, 2020), STITCH (http://stitch.embl.de/, updated September 26, 2020), Swiss Target Prediction (http://www.swisstargetprediction.ch/, updated September 26, 2020), and ChEMBL (https://www.ebi.ac.uk/chembl/, updated September 26, 2020). These genes were input into the Uniprot (https://www.uniprot.org/, updated September 28, 2020) database and set to “Homo sapiens” to ensure accurate target harvesting.

### Collection of candidate genes associated with COVID-19

To collect targets affiliated with COVID-19, the Human Gene Database (GeneCards, https://www.genecards.org/, updated September 25, 2020) and Online Mendelian Inheritance in Man (OMIM, https://www.omim.org/, updated September 25, 2020) were chosen. The whole primary genes or targets of emodin and COVID-19 were identified and subjected to intersection analysis *via* an online platform in the form of Venn diagrams (http://bioinformatics.psb.ugent.be/webtools/Venn/) to display the correlation between emodin-activated genes and their targets against COVID-19.

### Establishment of PPI network

The selected therapeutic targets of emodin against COVID-19 were input into the STRING (Version: 11.0, https://string-db.org/) database for PPI network analysis. Furthermore, the Cytoscape V3.7.2 plugin Network Analyzer was applied to analyze the network topology data through a series of parameters, such as degree centrality in the network. As a result, core targets were selected based on degree values. For filtering, the upper limit chosen was the maximum degree-value in the topology data, while the lower limit was the median of freedom as previously reported [20].

### Gene Ontology (GO) and Kyoto encyclopedia of genes and genomes (KEGG) pathway enrichment analysis

The potential core targets of emodin against COVID-19 were imported into the Metascape (http://metascape.org) database [21] by entering the list of target gene names and selecting “Homo sapiens” as the species for GO enrichment and KEGG pathway analysis. Go enrichment analysis generally includes three ontologies: biological process (BP), cellular component (CC), and molecular function (MF). To perform GO enrichment and KEGG pathway analysis, the statistical significance threshold was set at *P* < 0.01, and the WeChat online mapping website was used to visualize the analysis results.

### Verification of interactions between targets and emodin through molecular docking

The three-dimensional structure of emodin was downloaded from the PubChem database [22]. Subsequently, the MOL2 file was transformed into a PDB format using Open Babel 2.4.1 software. Then, we converted the PDB file into a pdbqt format using the AutoDock Tools v.1.5.6 software. The receptor proteins, including 3CL (PDB ID: 6LU7) and ACE2 (PDB ID:2AJF), were obtained from RCSB Protein Data Bank (PDB, http://www.rcsb.org/) database [23]. During the docking process, the proteins were imported into PyMOL 2.3.2 software to remove water molecules, and the Autodock Tools v.1.5.6 software was used to conduct hydrogenation and to calculate Gasteiger charges; these results were subsequently saved as the pdbqt format. The parameters of the receptor protein docking sites were set to cover those active pocket sites in which small molecular ligands complex might bind. In addition, the ligand compound (emodin) was set to flexibility and the receptor to rigidity. Furthermore, AutoDock Vina v.1.1.2 [24] was used to dock the compound and core targets or proteins, including the proteins of 3CL and ACE2 in SARS-CoV-2. The visualization of the docking results with the lowest binding energy was presented using PyMOL 2.3.2 software and LigPlot +v2.2.

## RESULTS

### Screening of the genes of emodin and COVID-19

**Figure 2:**
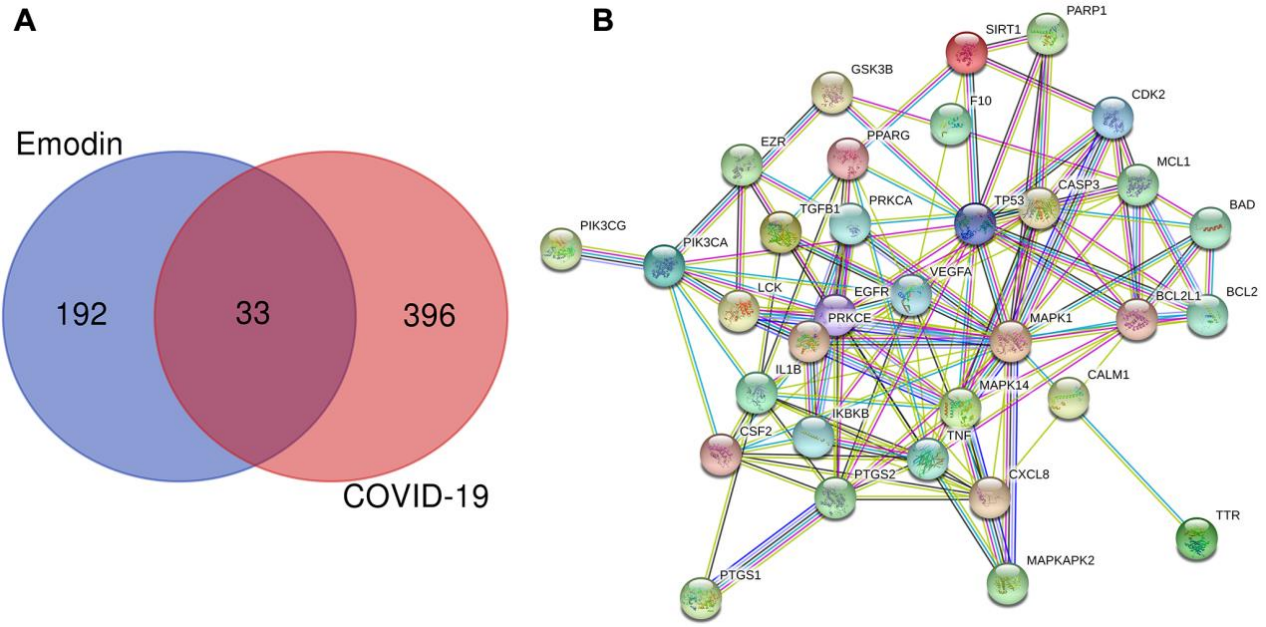
(**A**) Venn diagram was applied to present all candidate targets of emodin and COVID-19. (**B**) PPI network visualization of the potential therapeutic targets of emodin against COVID-19. The network nodes depict target proteins, and the edges represent protein-protein relationships.

To determine the genes associated with COVID-19 and the therapeutic targets of emodin, a series of bioinformatics analyses were carried out. We screened and identified a total of 429 COVID-19-associated genes from the Genecards and OMIM databases. Meanwhile, we also identified 225 pharmacodynamic targets of emodin from TCMSP, Drugbank, STITCH, Swiss Target Prediction, and ChEMBL databases. Subsequently, the aforementioned 429 targets were combined with these 225 targets of emodin, and a total of 33 potential therapeutic targets of emodin against COVID-19 were revealed (Figure 2A). Then the common genes were established in a PPI network diagram (Figure 2B) and we analyzed the PPI relationships using the STRING database.

**Figure 3:**
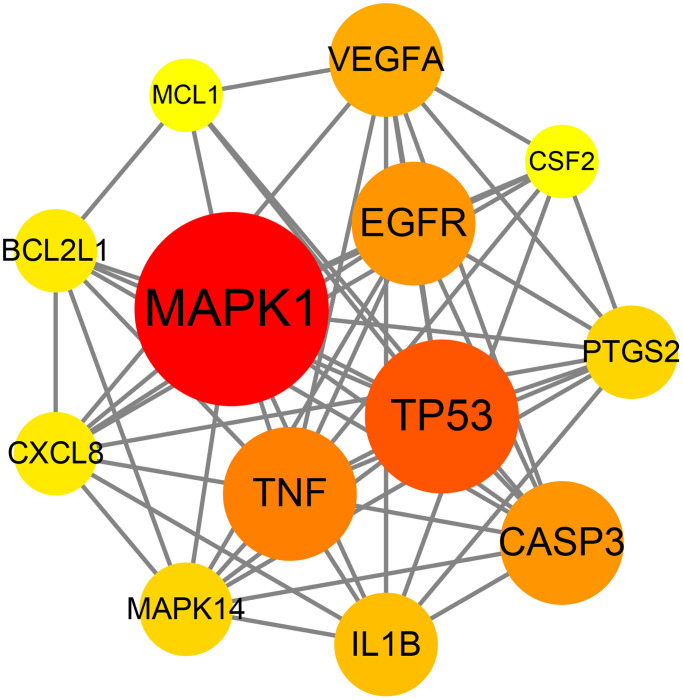
Network diagram of the core targets of emodin against COVID-19.

The data of the above PPI network were input into Cytoscape V3.7.2 software to acquire the information of the topology parameters. The results showed that the median degree of freedom was 8, and the maximum degree of freedom was 21. As shown in Figure 3, the top 13 targets of emodin against COVID-19, including mitogen-activated protein kinase 1 (MAPK1), tumor protein (TP53), tumor necrosis factor (TNF), caspase-3 (CASP3), epidermal growth factor receptor (EGFR), vascular endothelial growth factor A (VEGFA), interleukin 1B (IL1B), mitogen-activated protein kinase 14 (MAPK14), prostaglandin-endoperoxide synthase 2 (PTGS2), B-cell lymphoma-2-like protein 1 (BCL2L1), interleukin-8 (CXCL8), myeloid cell leukemia 1 (MCL1), and colony stimulating factor (CSF2), had a higher number of connections than other genes and were identified as core targets.

### Analysis of biological features of emodin against COVID-19

**Figure 4:**
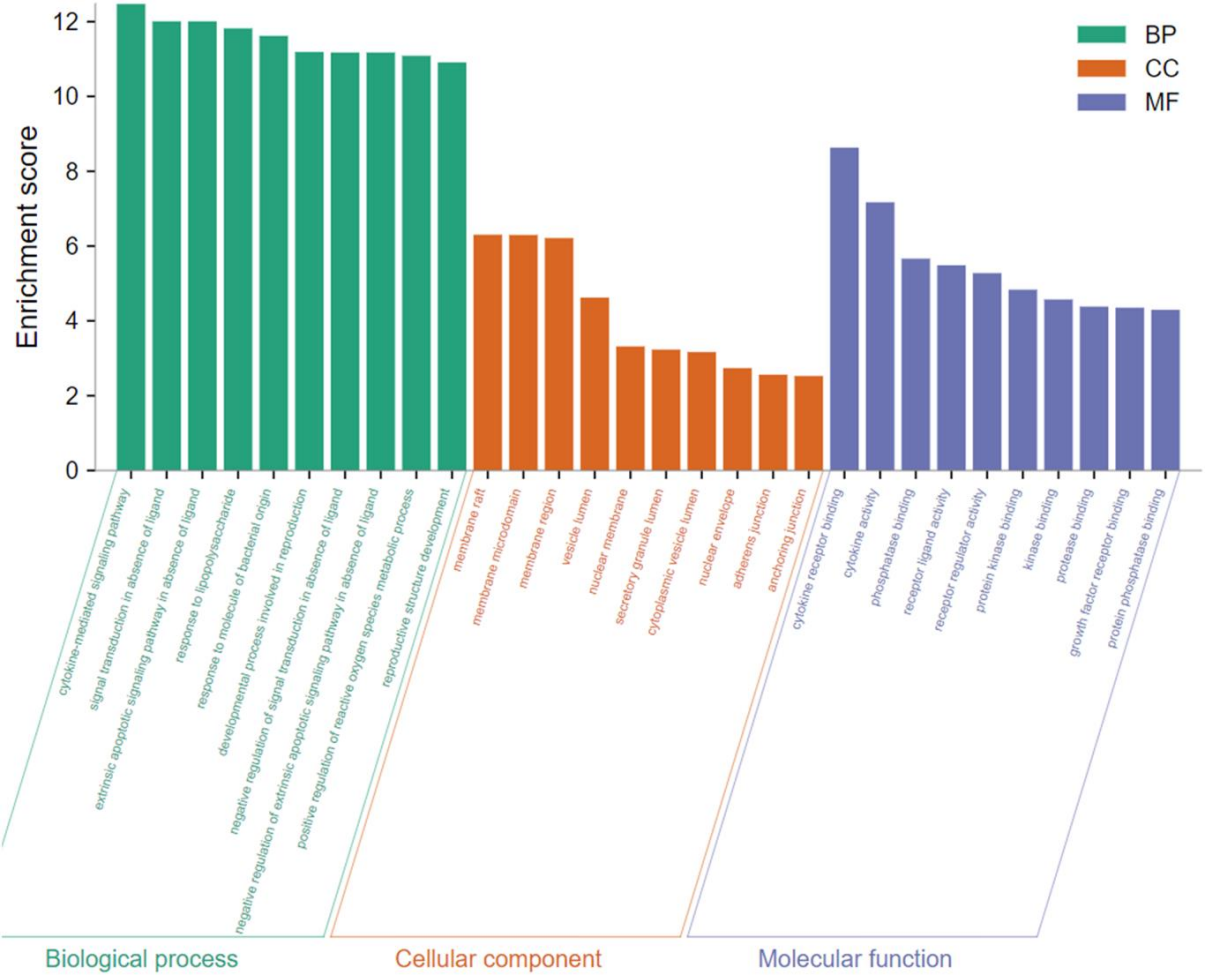
The top 10 of biological process, cellular component, and molecular function from GO terms analysis were illustrated by bar diagrams with enrichment scores (the x-axis represents top 10 GO terms, and the y-axis indicates the *p*-value).

**Figure 5:**
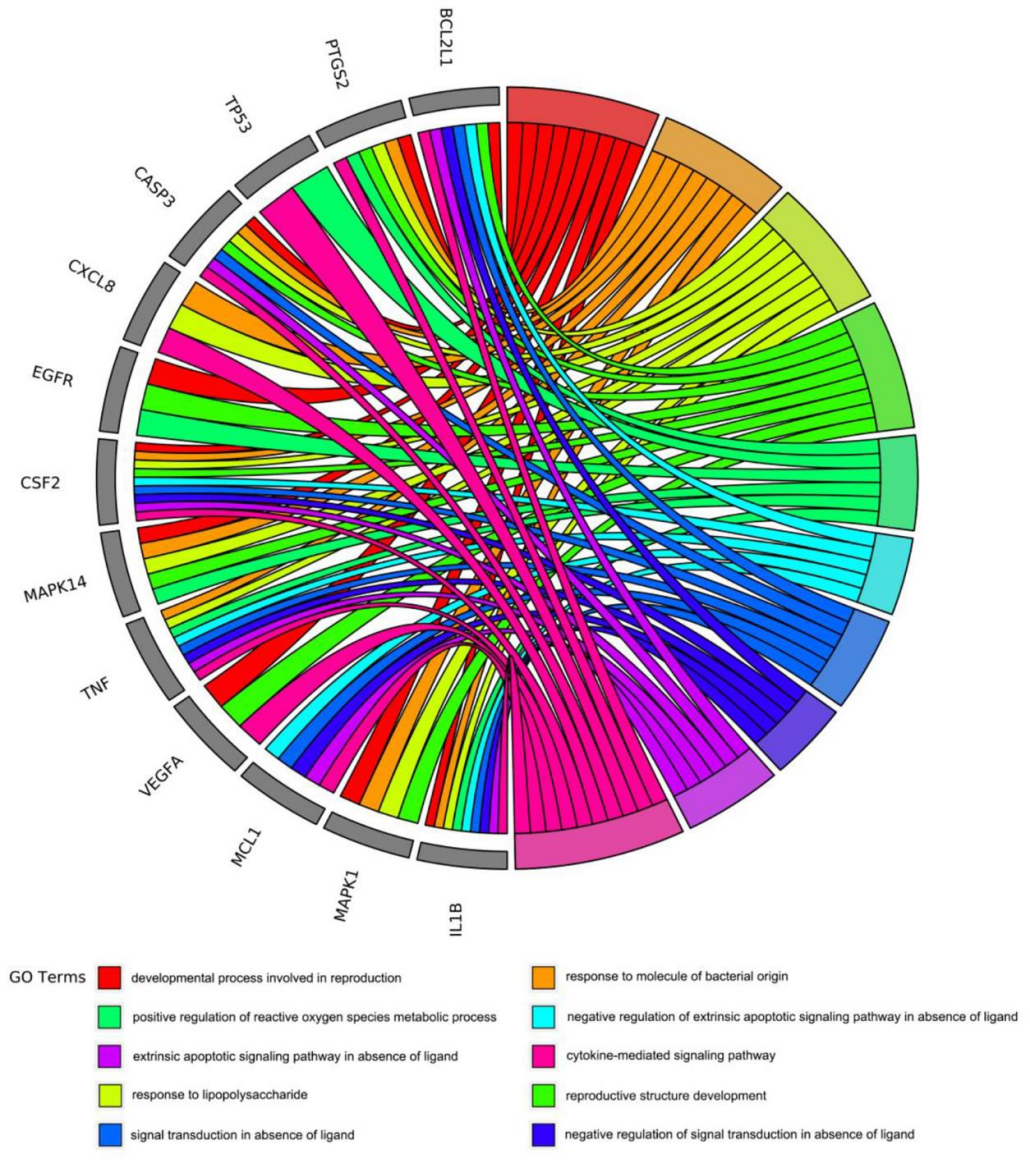
The core targets of emodin-treated COVID-19 were linked to the top 10 most enriched GO BP terms in GO chord plot.

To gain insight into the potential biological features and signaling pathways of emodin against COVID-19, we performed functional annotation of the core targets based on GO enrichment and KEGG pathway analysis. Total 543 GO terms were selected, of which BP accounted for 514, CC for 11, and MF for 18. As shown in Figure 4, among them, most GO terms associated with the BP category were correlated with cytokine-mediated signaling pathway, signal transduction in absence of ligand, extrinsic apoptotic signaling pathway in the absence of ligand, response to lipopolysaccharide, response to molecule of bacterial origin, developmental process involved in reproduction, reproductive structure development, etc. The CC enrichment analysis mainly included the following concepts: membrane raft, membrane microdomain, membrane region, vesicle lumen, nuclear membrane, secretory granule lumen, cytoplasmic vesicle lumen, nuclear envelope, adherens junction, anchoring junction, etc. Simultaneously, MF annotation mainly presented cytokine receptor binding, cytokine activity, phosphatase binding, receptor-ligand activity, receptor regulator activity, protein kinase binding, kinase binding, protease binding, growth factor receptor binding, protein phosphatase binding, etc. Interestingly, as shown in Figure 5, the core targets of emodin against COVID-19 were directly related with the top 10 terms of the GO BP category, while the top 10 terms of the BP category had many targets in common, suggesting that the core targets of emodin may be involved in various regulatory processes of BP to resist COVID-19 effects.

### Analysis of emodin-mediated signaling pathway and network against COVID-19

**Figure 6:**
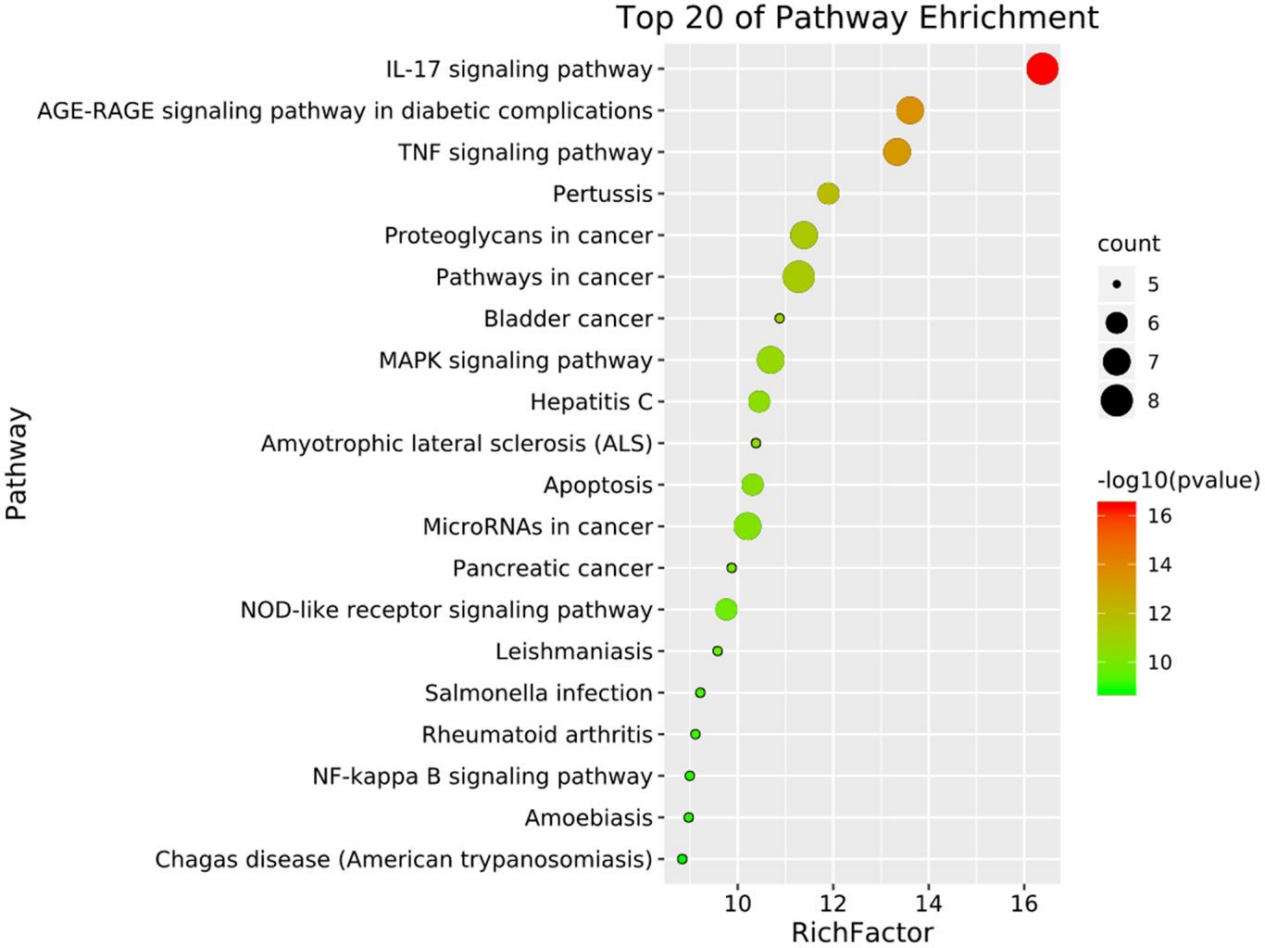
**The top 20 signaling pathways from KEGG enrichment analysis were showed by the bubble diagram with count algorithms and *p*-values.** Each node signals one KEGG pathway, and its size represents the gene number. The color indicates the *p*-value.

**Figure 7:**
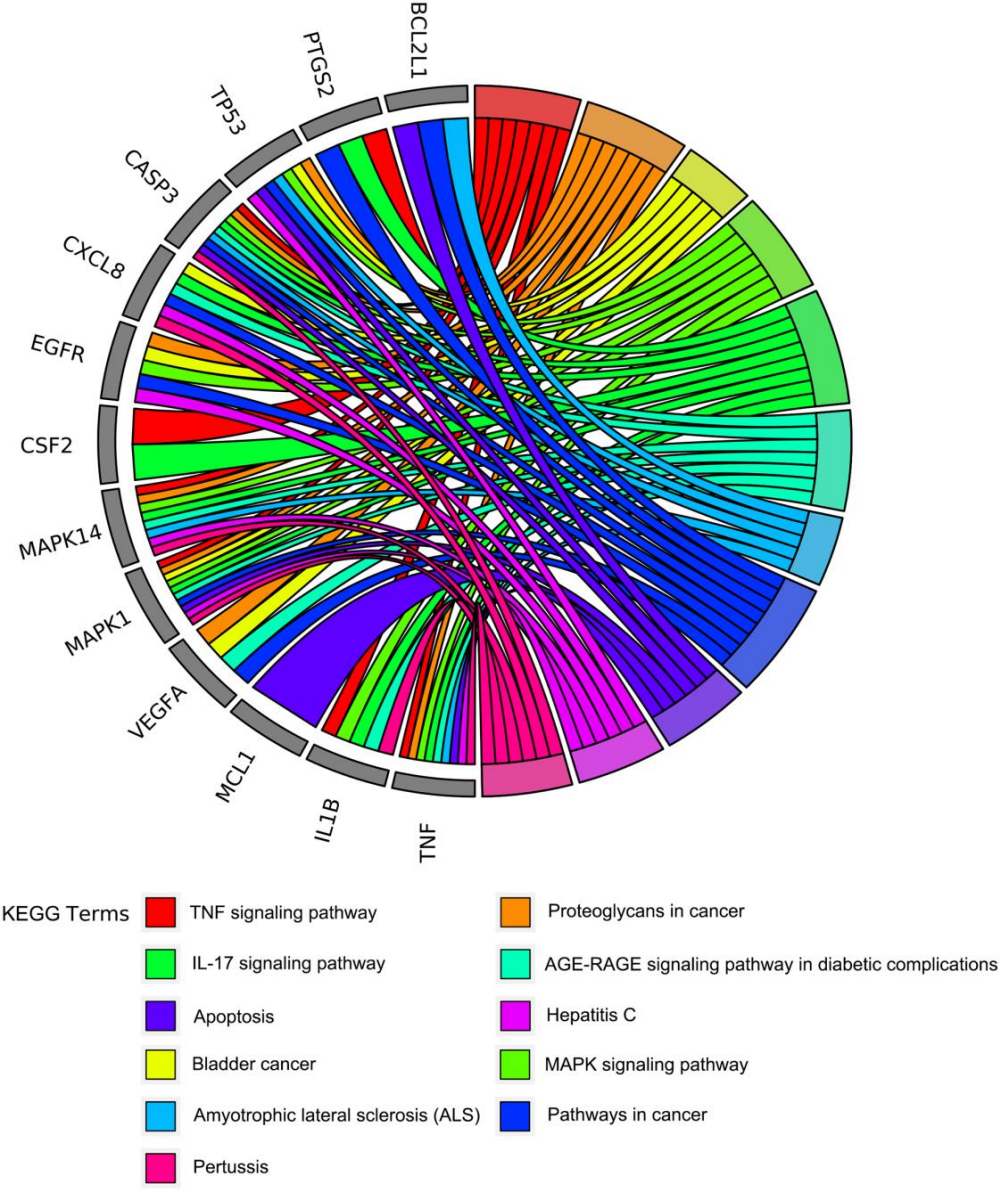
**The circo-diagrams of the top 11 ranked overrepresented KEGG pathways.** Different colors on the right side of the graph represent different signaling pathways, and the left side is the top 13 core targets of emodin against COVID-19 with relevance.

**Figure 8:**
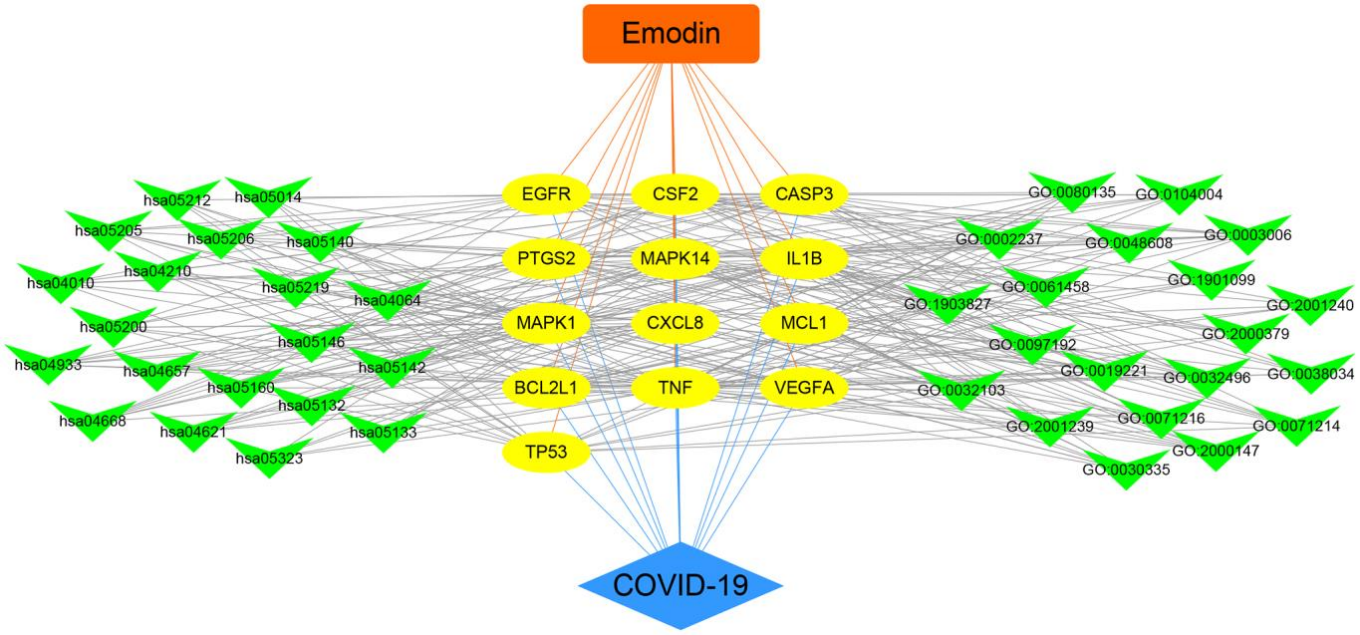
Integrated visualization of interaction network of target-disease-function-pathway in emodin-treated COVID-19.

To further understand the potential mechanisms of emodin against COVID-19, KEGG pathway enrichment was carried out. From data analysis, a total of 72 pathways were enriched based on the adjusted *p*-value parameter (*P* < 0.01). The KEGG pathway results demonstrated that the main pathways included IL-17 signaling pathway, AGE-RAGE signaling pathway in diabetic complications, TNF signaling pathway, pertussis, proteoglycans in cancer, and other contagious pulmonary diseases. Apart from the above, we also found that emodin could mediate many signaling pathways, including MAPK signaling pathway, NOD-like receptor signaling pathway, NF-kappa B signaling pathway, as well as many pathways related to inflammatory or immune responses (Figure 6). As shown in Figure 7, thirteen core targets of emodin against COVID-19 were directly associated with the top 11 pathways, indicating that the core targets of emodin could be treatments of COVID-19 by acting on multiple pathways. We analyzed the potential mechanisms in the treatment of COVID-19. Based on network pharmacology, we established the interaction network diagram of “emodin-target-GO-KEGG-COVID-19” by the application of Cytoscape V3.7.2 software (Figure 8).

Subsequently, we created network diagrams which highlighted core targets of KEGG-enriched pathways using the ‘pathview’ package in *R* for emodin against COVID-19 (Supplementary Figure 1–5).

### Results of molecular docking

**Figure 9:**
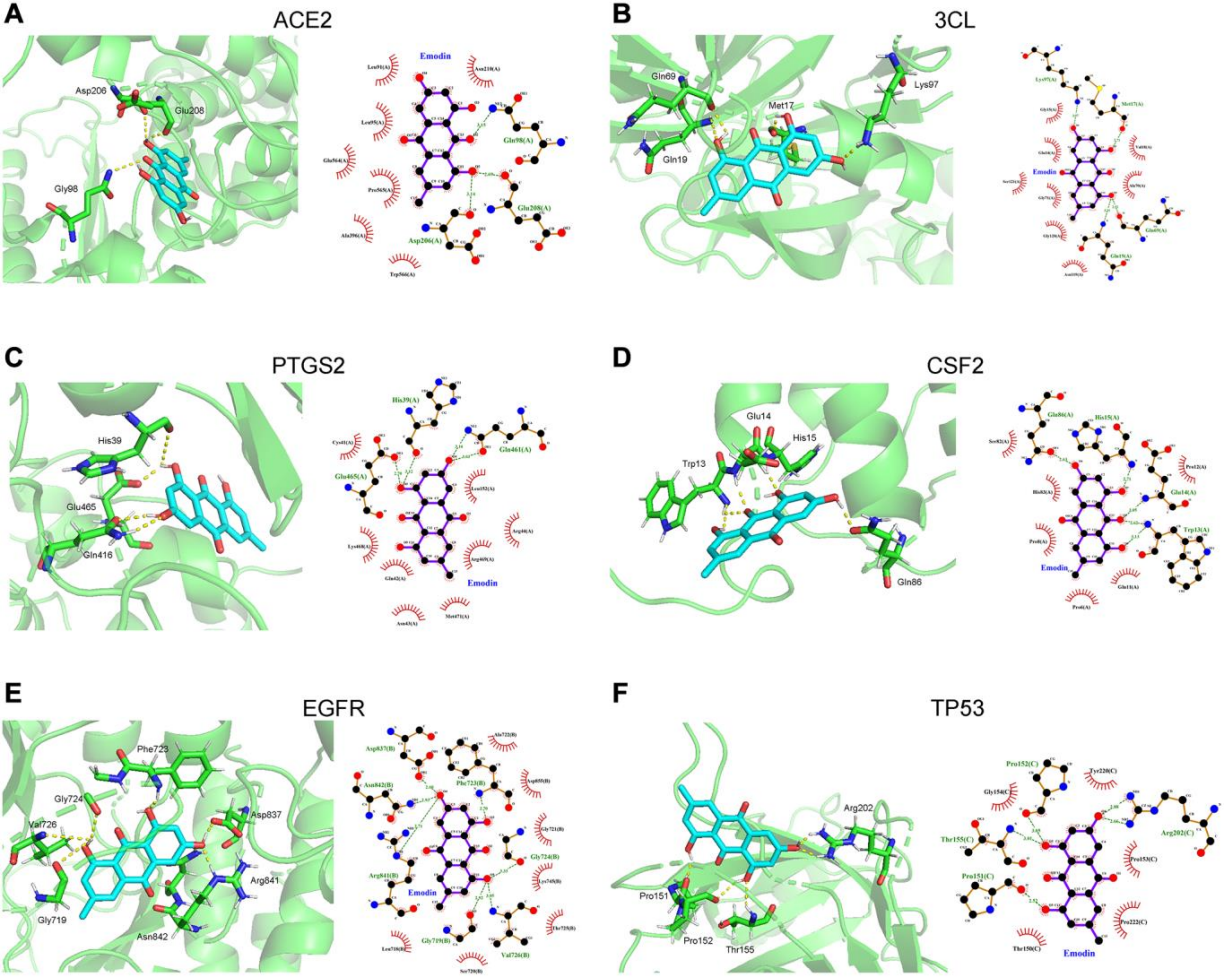
**Molecular docking diagram showed that the binding capability of emodin against COVID-19 was presented as 3D diagrams and 2D diagrams, respectively.** (**A**) Angiotensin converting enzyme 2 (ACE2), (**B**) 3CL. (**C**) Prostaglandin endoperoxide synthase 2 (PTGS2). (**D**) Colony stimulating factor 2 (CSF). (**E**) Epidermal growth factor receptor (EGFR). (**F**) Tumor protein 53 (TP53).

Molecular docking analysis predicted the binding capability between emodin and the 15 tested target proteins (Table 1). In this analysis, the value of the docking score indicates the binding activity between the compound and the protein. The lower the docking score, the more stable the binding conformation, and the greater the possibility of molecular interaction. Based on a binding energy of less than -7.0 kcal/mol, ACE2, 3CL, PTGS2, CSF2, EGFR, and TP53 of the 15 tested target proteins were selected. As indicated in Figure 9, in terms of interaction points, emodin mainly interacted with amino acid residues Asp206, Glu208 and Gly98 in ACE2. Meanwhile, emodin mainly interacted with 3CL via Gln19, Gln69, Met17 and Lys97 (amino acid residues) and could bind to PTGS2 amino acid residues Gln461, Glu465 and His39. Also, emodin mainly interacted with CSF2 bridged with amino acid residues (Trp13, Glu14, His15, and Gln86). In the EGFR protein, emodin formed hydrogen bonds with amino acid residues Val726, Gly724, Phe723, Asp837, Arg841, Asn842 and Gly719. Finally, the hydrogen bonding between emodin and the TP53 protein acted on the amino acid residues Arg202, Thr155, Pro151 and Pro152.

**Table 1:** Binding energy for core targets.

**Targets**	**Binding energy (kcal/mol)**	**Targets**	**Binding energy (kcal/mol)**
MAPK1	−6.87	PTGS2	−8.51
TP53	−7.03	BCL2L1	−5.77
TNF	−6.46	CXCL8	−5.80
CASP3	−6.96	MCL1	−5.99
EGFR	−7.65	CSF2	−7.97
VEGFA	−6.10	3CL	−7.04
IL1B	−6.97	ACE2	−7.26
MAPK14	−6.69		

## DISCUSSION

With the outbreak of COVID-19, more attention to the transmission and effects of SARS-CoV-2 has been paid worldwide. Confirmed cases of COVID-19 have sharply increased in number over time despite the novelty of the causative virus. Prior works by researchers have reported that although emodin possesses potential efficacy against the SARS virus [15], the mechanism of emodin against COVID-19 is not clear. Herein, we revealed a pharmacological mechanism of emodin against COVID-9 using integrative network pharmacology and other bioinformatics technologies. Our results revealed that the top 20 pathways were mainly involved in viral infections, inflammation, immune responses, cancer, and other contagious pulmonary diseases. Thus, we could speculate that emodin may contribute to the treatment of COVID-19 *via* regulating inflammation-related pathways, immune regulation-related pathways, and viral infection-related pathways. Moreover, in the PPI network, the top 13 targets, including BCL2L1, PTGS2, TP53, CASP3, CXCL8, EGFR, CSF2, MAPK14, TNF, VEGFA, MCL1, MAPK1, and IL1B, were selected as core targets that may play vital roles in emodin against COVID-19. These core targets were enriched in the different signaling pathways. We obtained some pathways related to immune responses, bacterial structure and reproduction, inflammatory response diabetic complications, and cancers based on GO enrichment and KEGG pathway analysis.

Cytokine-mediated signaling pathway can participate in severe inflammatory responses, which may lead to pneumonia in people with COVID-19 [25]. Due to the release of tissue factor in response to cytokines triggered by endothelial cells, it may further progress to the formation of coagulation in COVID-19 patients [26]. In addition, lipopolysaccharides cause inflammatory effects and are also pro-inflammatory compounds of bacterial origin [27]. Importantly, the function of lipopolysaccharide or molecule of bacterial origin provides viruses with thermal stabilization [28]. The reproduction number is an important parameter by which transmissibility can be measured during epidemics [29]. A prior study by Khajanchi et al. [30] reported a higher value, suggesting the possibility of an outbreak of COVID-19 phenomena in India. Administration of emodin may facilitate the host to induce anti-inflammatory response to achieve anti-COVID-19 effects.

Our KEGG pathway analysis uncovered that these pathways were correlated with immune response, inflammatory response, and the recognition of pathogen-associated molecular modes. KEGG pathway analysis exhibited that some related signaling pathways were affected by emodin in the treatment of COVID-19, including IL-17 signaling pathway, MAPK signaling pathway, NF-kappa B signaling pathway, NOD-like receptor signaling pathway, and TNF signaling pathway, as presented by KEGG pathway analysis (Supplementary Figure 1–5). The IL-17 signaling pathway is critical for clearing extracellular pathogens and is associated with acute respiratory distress syndrome [31]. One study demonstrated that there is the elevation of IL-17 in bronchoalveolar lavage fluid in patients with acute respiratory distress syndrome [32]. Another study showed that the limitation of IL-17 signaling can inhibit the lung inflammation in mice [33], indicating a potential mechanism in the treatment of COVID-19 *via* the reduction of IL-17 levels. Meanwhile, the activation of IL-17 can enhance the expression of cytokines [34], which can be found in patients with COVID-19. The downstream signaling of IL-17 is linked to NF-kappa B signaling and MAPK signaling. IL-17 also boosts the generation of chemokines and develops the recruitment of immune cells [35, 36]. Other researchers have found that the expression levels of several inflammatory factors are increased in patients with COVID-19 by way of a quantitative proteomics approach combined with bioinformatics analyses, which is possibly caused by the activation of NF-kappa B signaling [37]. Similarly, MAPK signaling inactivation contributes to a reduced the chance of COVID-19 [38, 39]. Prior studies have suggested that elevated pro-inflammatory IL-17 is associated with elevated NOD-like receptor [40, 41]. NOD-like receptor, one of the inflammatory components, can increase the expression of interleukin- 1β [35]. Interleukin can regulate the immune response and is involved in the inflammatory response. Apart from the above, elevated NOD-like receptor genes lead to the degradation of the viral genome and inhibition of the virus [42].

In addition, patients with COVID-19 also have many complications, such as diabetes and cancer. ACE2, the SARS-CoV-2 receptor, can increase the risk of COVID-19 in patients with diabetes in clinic [43]. Also, there is a clue that ACE2-positive expression in diabetic disease was observed in SARS-CoV-2 infected cells [44]. These findings support that emodin administration may improve the symptoms of COVID-19 *via* regulating targets related to diabetes.

Molecular docking presents a tool that contributes to the discovery or design of drugs. According to the literature, AEC2 and 3CL proteins serve as direct targets for blocking COVID-19 [1]. Meanwhile, the proteins of CFS2 and PTGS2 are associated with inflammation [45], and EGFR and TP53 regulate the immune response [46]. Based on the aforementioned molecular docking results, the scores of emodin with the key targets were all less than −5.0 kcal/mol, of which 6 targets had a score less than −7.0 kcal/mol. Their interaction sites or points were uncovered, indicating that emodin had good binding activities with ACE2, 3CL, PTGS2, CSF2, EGFR, and TP53, respectively. Therefore, based on our data, emodin shows great potential for treating COVID-19. Although its efficacy still needs to be verified by further experimental work.

## CONCLUSIONS

Briefly, network pharmacology revealed all core targets, biological function, and signaling pathways of emodin against COVID-19. The present results showed that emodin could contribute to treating COVID-19 *via* the regulation of inflammation-related pathways, immune regulation-related pathways, and viral infection-related pathways. Furthermore, these molecular docking results indicated the potential benefits for supporting the possibility of emodin-treated COVID-19 in clinic. According to our data, emodin may serve as a promising candidate drug for the treatment for COVID-19.

## Supplementary Materials

Supplementary Figures
