# Supplementary material for: Revealing the therapeutic targets and molecular mechanisms of emodin-treated coronavirus disease 2019 via a systematic study of network pharmacology
Source: Aging (Albany NY). 2021 Jun 4;13(11):14571–89. doi: 10.18632/aging.203098 (PMC8221358; doi:10.18632/aging.203098)
Supplement: Supplementary Figures [file aging-13-203098-s001.pdf]

SUPPLEMENTARY FIGURES

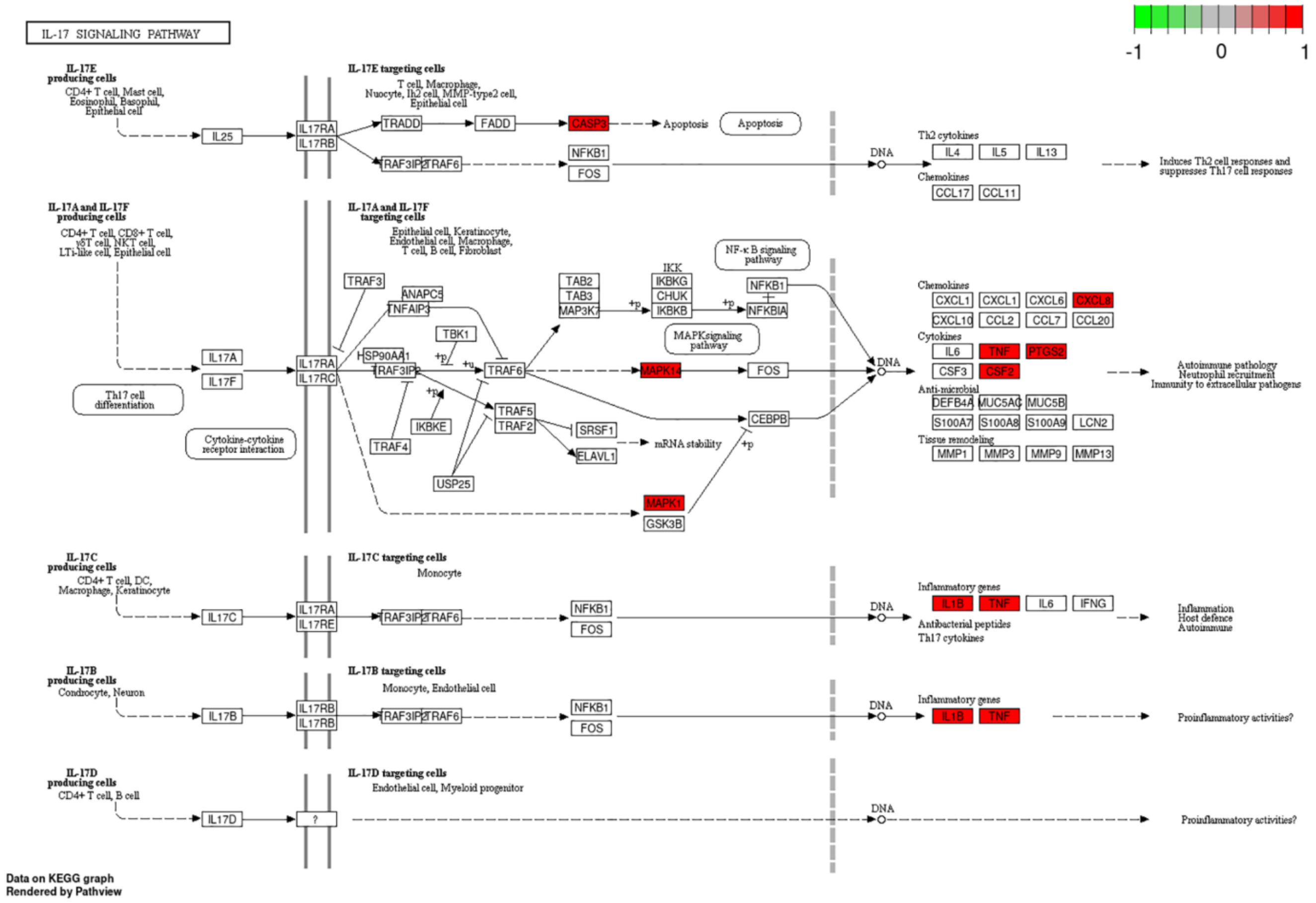

Supplementary Figure 1. IL-17 signaling pathway as one of the main pathways related to the therapeutic mechanism of emodin.

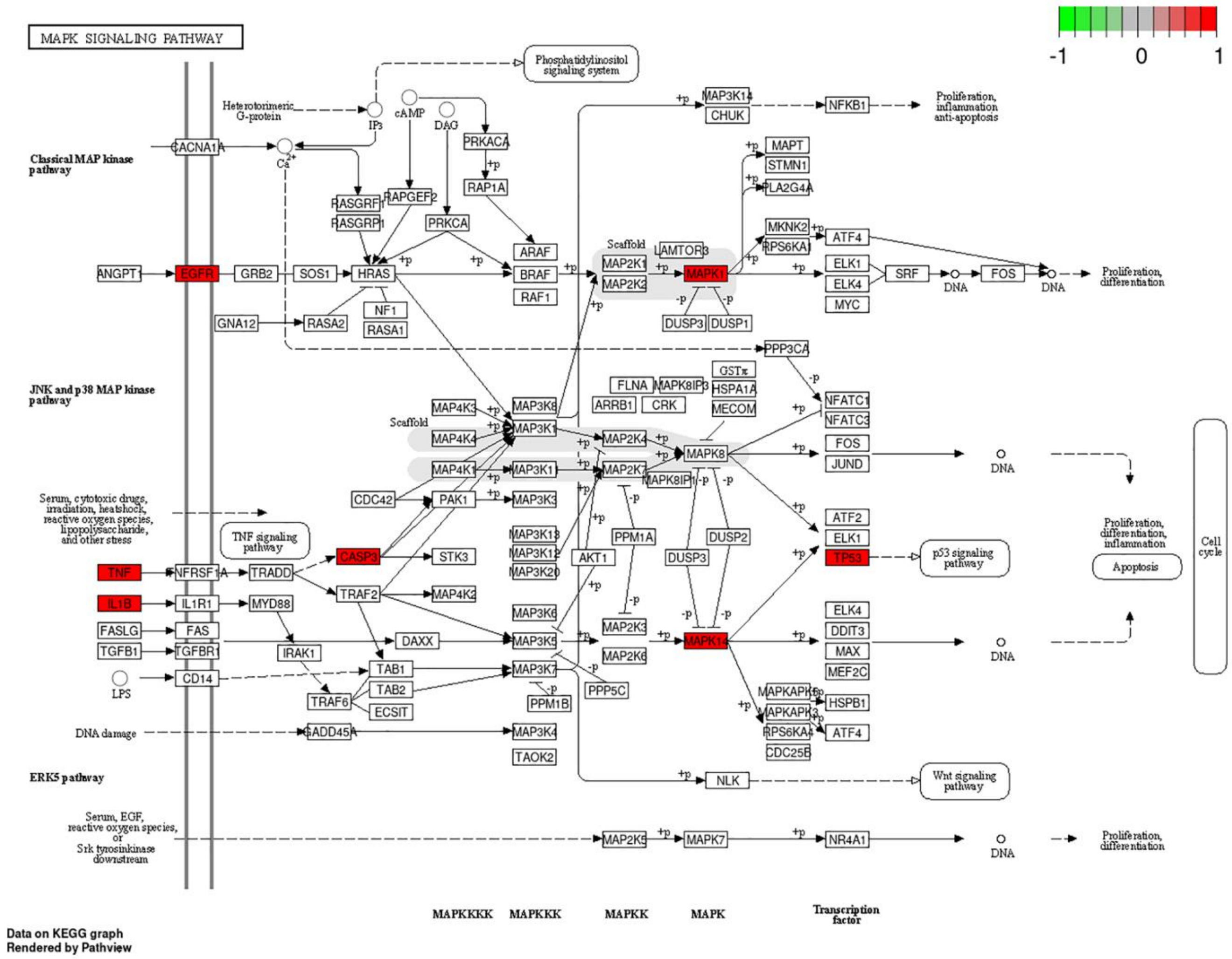

Supplementary Figure 2. MAPK signaling pathway as one of the main pathways related to the therapeutic mechanism of emodin.

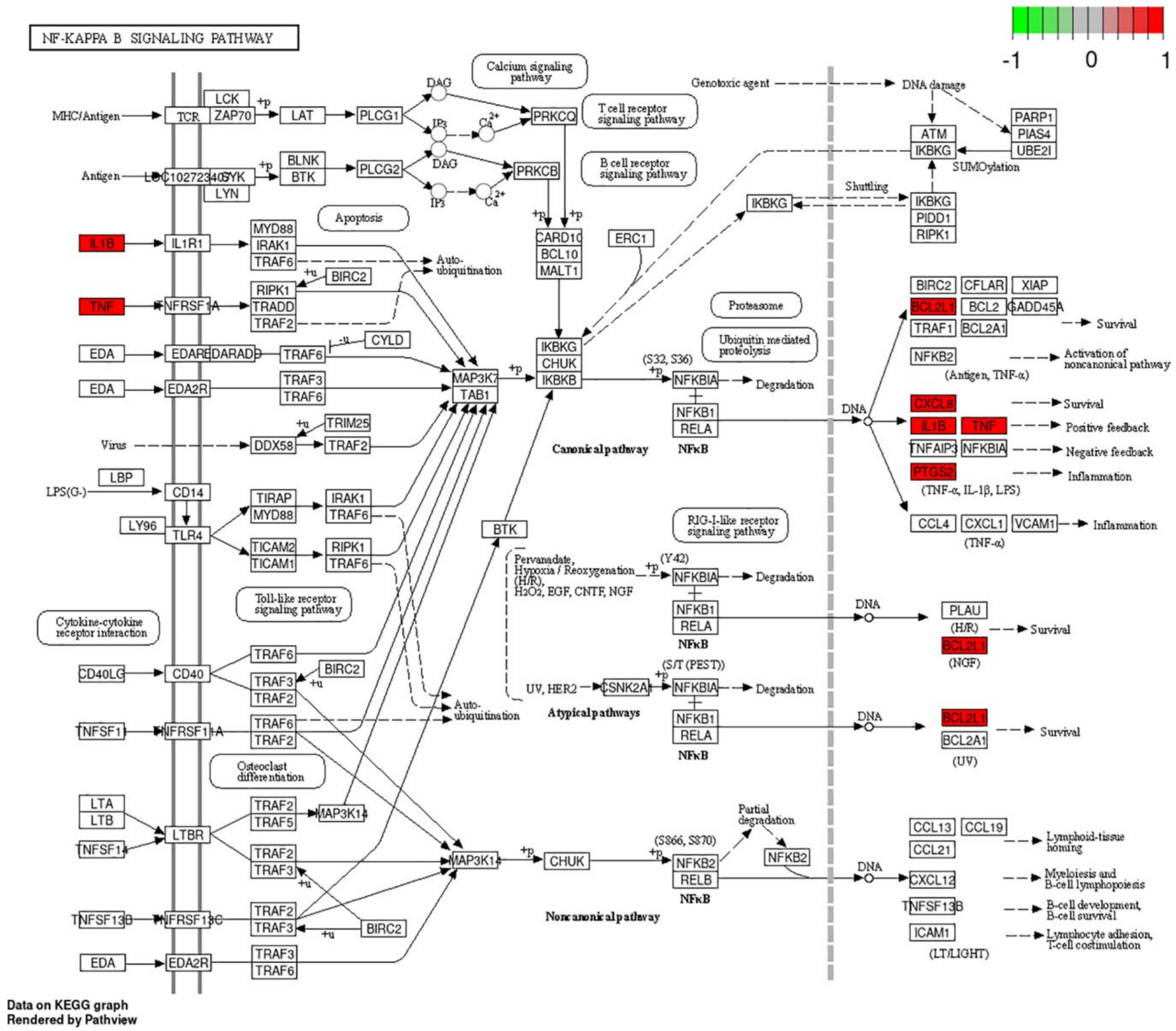

Supplementary Figure 3. NF-kappa B signaling pathway as one of the main pathways related to the therapeutic mechanism of emodin.

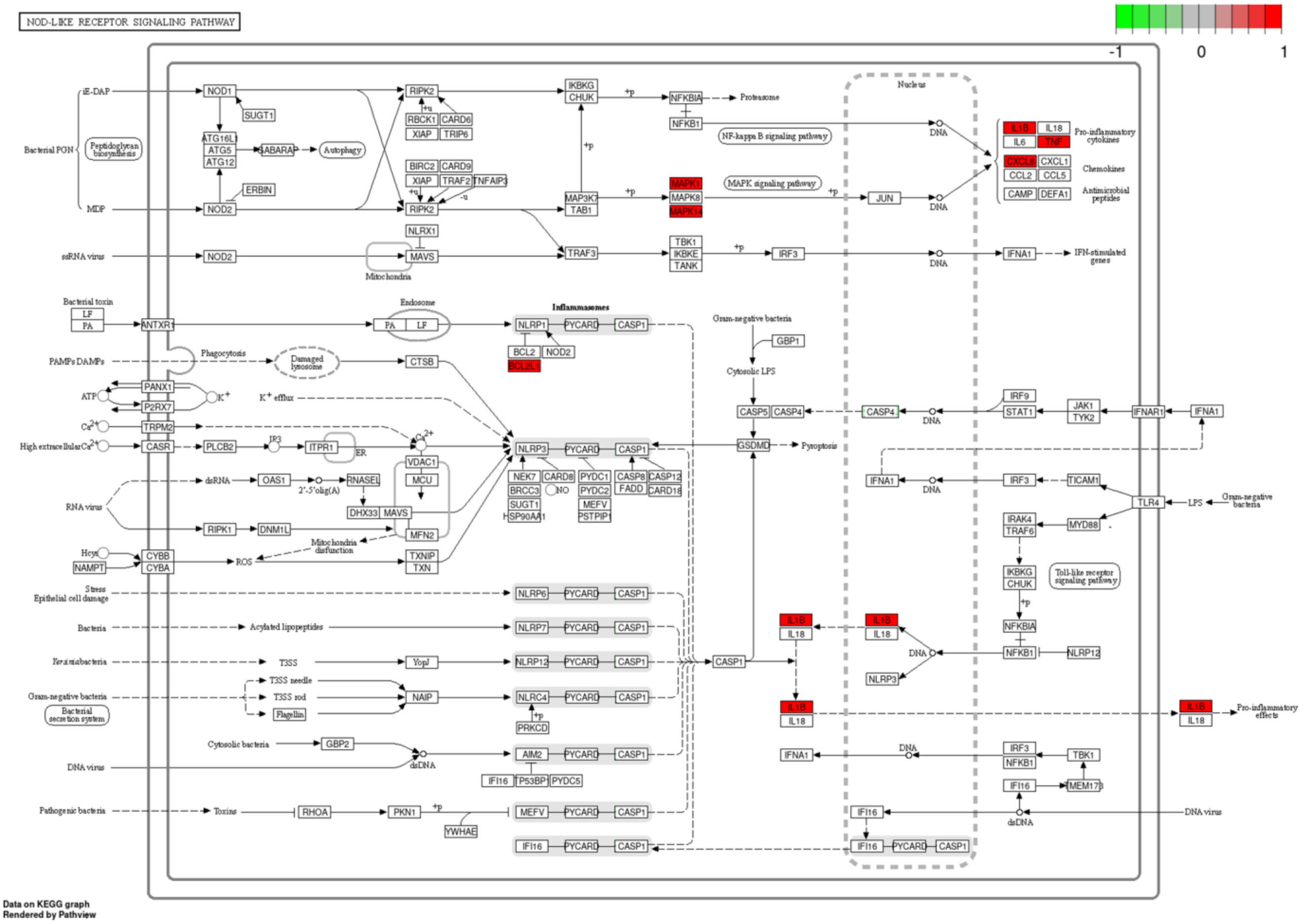

Supplementary Figure 4. NOD-like receptor signaling pathway as one of the main pathways related to the therapeutic mechanism of emodin.

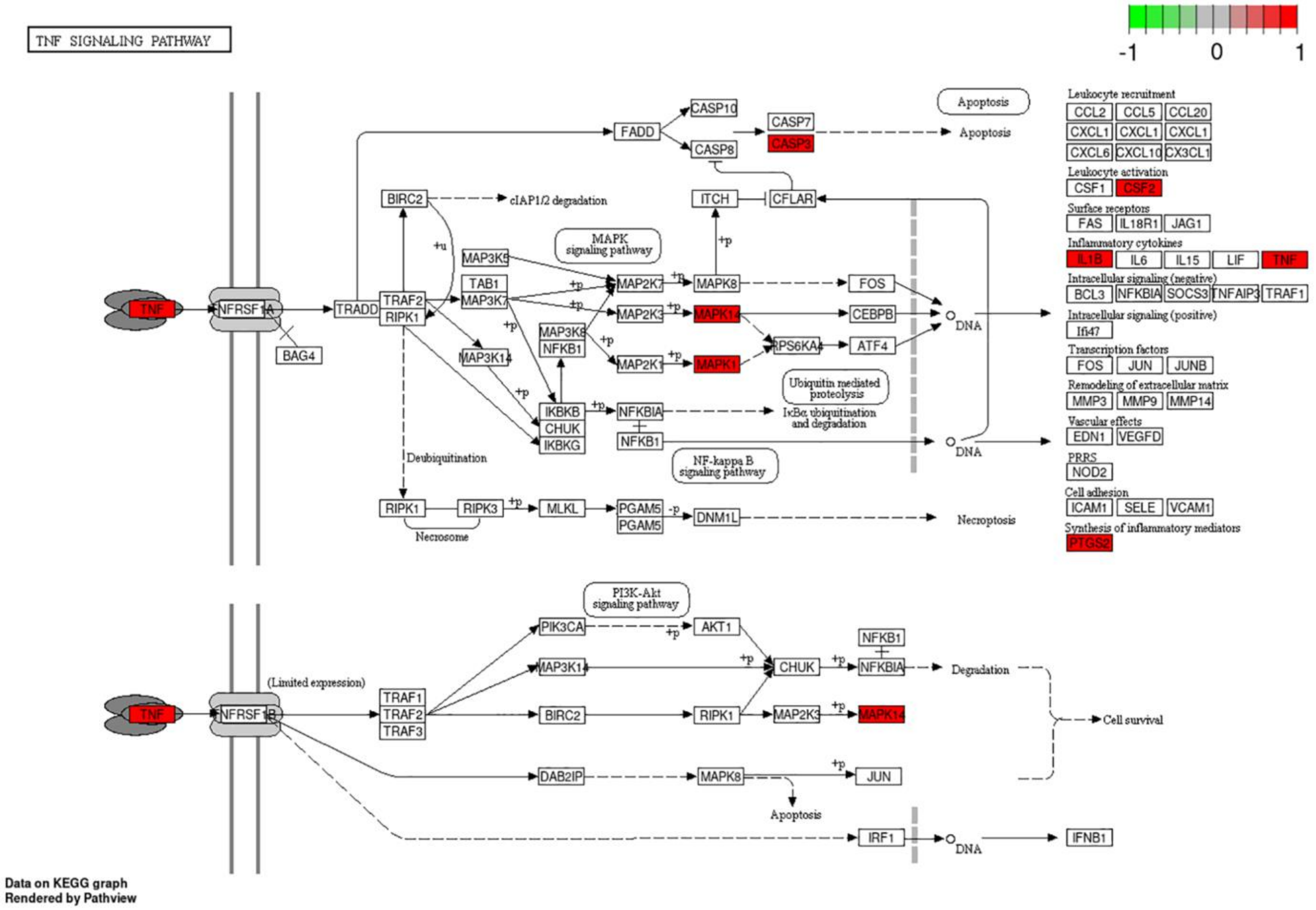

Supplementary Figure 5. TNF signaling pathway as one of the main pathways related to the therapeutic mechanism of emodin.
